# Testing statistical significance scores of sequence comparison methods with structure similarity

**DOI:** 10.1186/1471-2105-7-444

**Published:** 2006-10-12

**Authors:** Tim Hulsen, Jacob de Vlieg, Jack AM Leunissen, Peter MA Groenen

**Affiliations:** 1 Centre for Molecular and Biomolecular Informatics (CMBI), Nijmegen Centre for Molecular Life Sciences (NCMLS), Radboud University Nijmegen Medical Centre, Nijmegen, The Netherlands; 2 Molecular Design and Informatics, NV Organon, Oss, The Netherlands; 3 Laboratory of Bioinformatics, Wageningen University and Research Centre, Wageningen, The Netherlands

## Abstract

**Background:**

In the past years the Smith-Waterman sequence comparison algorithm has gained popularity due to improved implementations and rapidly increasing computing power. However, the quality and sensitivity of a database search is not only determined by the algorithm but also by the statistical significance testing for an alignment. The e-value is the most commonly used statistical validation method for sequence database searching. The CluSTr database and the Protein World database have been created using an alternative statistical significance test: a Z-score based on Monte-Carlo statistics. Several papers have described the superiority of the Z-score as compared to the e-value, using simulated data. We were interested if this could be validated when applied to existing, evolutionary related protein sequences.

**Results:**

All experiments are performed on the ASTRAL SCOP database. The Smith-Waterman sequence comparison algorithm with both e-value and Z-score statistics is evaluated, using ROC, CVE and AP measures. The BLAST and FASTA algorithms are used as reference. We find that two out of three Smith-Waterman implementations with e-value are better at predicting structural similarities between proteins than the Smith-Waterman implementation with Z-score. SSEARCH especially has very high scores.

**Conclusion:**

The compute intensive Z-score does not have a clear advantage over the e-value. The Smith-Waterman implementations give generally better results than their heuristic counterparts. We recommend using the SSEARCH algorithm combined with e-values for pairwise sequence comparisons.

## Background

Sequence comparison is still one of the most important methodologies in the field of computational biology. It enables researchers to compare the sequences of genes or proteins with unknown functions to sequences of well-studied genes or proteins. However, due to a significant increase in whole genome sequencing projects, the amount of sequence data is nowadays very large and rapidly increasing. Therefore, pairwise comparison algorithms should not only be accurate and reliable but also fast. The Smith-Waterman algorithm [1] is one of the most advanced and sensitive pairwise sequence comparison algorithms currently available. However, it is theoretically about 50 times slower than other popular algorithms [2], such as FASTA [3] and BLAST [4]. All three algorithms generate local alignments, but the Smith-Waterman algorithm puts no constraints on the alignment it reports other than that it has a positive score in terms of the similarity table used to score the alignment. BLAST and FASTA put additional constraints on the alignments that they report in order to speed up their operation: only sequences above a certain similarity threshold are reported, the rest is used for the estimation of certain parameters used in the alignment calculation. Because of this Smith-Waterman is more sensitive than BLAST and FASTA. The Smith-Waterman algorithm finds the best matching regions in the same pair of sequences. However, BLAST and FASTA are still far more popular because of their speed and the addition of a statistical significance value, the Expect-value (or simply e-value), whereas the original Smith-Waterman implementation relies only on the SW-score without any further statistics. The newer Smith-Waterman implementations of Paracel [5], SSEARCH [6] and ParAlign [7] do include the e-value as a measure of statistical significance, which makes the Smith-Waterman algorithm more usable as the engine behind a similarity search tool. The e-value is far more useful than the SW-score, because it describes the number of hits one can expect to see by chance when searching a database of a certain size. An e-value threshold can be used easily to separate the 'interesting' results from the background noise. However, a more reliable statistical estimate is still needed [8]. The Z-score, based on Monte-Carlo statistics, was introduced by Doolittle [9] and implemented by Gene-IT [10] in its sequence comparison suite Biofacet [11]. The Z-score has been used in the creation of the sequence annotation databases CluSTr [12] and Protein World [13] and was used in orthology studies [14]. The Z-score has also been implemented in algorithms other than Smith-Waterman, such as FASTA [15]. It is calculated by performing a number (e.g., 100) of shuffling randomizations of both sequences that are compared, completed by an estimation of the SW score significance as compared to the original pairwise alignment. This makes the Z-score very useful for doing all-against-all pairwise sequence comparisons: Z-scores of different sequence pairs can be compared to each other, because they are only dependent on the sequences itself and not on the database size, which is one of the parameters used to calculate the e-value. However, this independency of the database size makes the Z-score unsuitable for determining the probability that an alignment has been obtained by chance. The randomizations make the Z-score calculation quite slow, but theoretically it is more sensitive and more selective than e-value statistics [16,17]. Unfortunately, this has never been validated experimentally.

Some methods have been used to combine the sensitivity and selectivity of a sequence comparison algorithm into one single score [18]. Receiver operating characteristic (ROC) is a popular measure of search accuracy [19]. For a perfect search algorithm, all true positives for these queries should appear before any false positive in the ranked output list, which gives an ROC score of 1. If the first n items in the list are all false positives, the ROC_n _score is 0. Although researchers have devised many ways to merge ROC scores for a set of queries [20], one simple and popular method is to 'pool' search results so as to get an overall ROC score [21]. Another method to evaluate different methods is the errors per query (EPQ) criterion and the 'coverage versus error' plots [2]. EPQ is a selectivity indicator based on all-against-all comparisons, and coverage is a sensitivity measure. The assumption for EPQ is that the search algorithm can yield a 'normalized similarity score' rather than a length-dependent one, so that results from queries are comparable. Like ROC, the coverage versus error plot can give an overall performance comparison for search algorithms. A third method, the average precision (AP) criterion, is adopted from information retrieval research [22]. The method defines two values: the recall (true positives divided by the number of homologs) and the precision (true positives divided by the number of hits), which are plotted in a graph. The AP then is an approximate integral to calculate the area under this recall-precision curve. These methods were used to compare several sequence comparison algorithms, but we use them to compare the e-value and Z-score statistics. Analyses of BLAST and FASTA are also included as reference material.

Here we show that two out of the three Smith-Waterman implementations with e-value statistics are more accurate than the Smith-Waterman implementation of Biofacet with Z-score statistics. Furthermore, the comparison of BLAST and FASTA with the four Smith-Waterman implementations shows that FASTA is a more reliable algorithm when using the ASTRAL SCOP structural classification as a benchmark. The Smith-Waterman implementation of Paracel even has lower scores than both BLAST and FASTA. SSEARCH, the Smith-Waterman implementation in the FASTA package, scores best.

## Results

We used a non-redundant protein-domain sequence database derived from PDB as the target database. It is automatically generated using the ASTRAL system [23]. According to the structural classification of proteins (SCOP release 1.65), it includes 9498 sequences and 2326 families. True positives are those in the same family as the query sequence. SCOP as an independent and accurate source for evaluating database search methods has been used by other researchers [2,24]. ASTRAL SCOP sets with different maximal percentage identity thresholds (10%, 20%, 25%, 30%, 35%, 40%, 50%, 70%, 90% and 95%) were downloaded from the ASTRAL SCOP website [25]. Their properties (number of families, number of members, etc.) are shown in table 1. Three different statistical measures were applied: receiver operating characteristic (ROC), coverage versus error (CVE) and mean average precision (AP). We compared six different pairwise sequence comparison algorithms, which are listed in table 2, together with the parameters used in this study.

**Table 1:** Properties of ASTRAL SCOP PDB sets

**Maximal percentage indentity**	**Number of sequences**	**Number of families**	**Average family size**	**Size of largest family**	**Number of families having only 1 member**	**Number of families having more than 1 member**
**10%**	3631	2250	1.614	25	1655	595
**20%**	3968	2297	1.727	29	1605	692
**25%**	4357	2313	1.884	32	1530	783
**30%**	4821	2320	2.078	39	1435	885
**35%**	5301	2322	2.283	46	1333	989
**40%**	5674	2322	2.444	47	1269	1053
**50%**	6442	2324	2.772	50	1178	1146
**70%**	7551	2325	3.248	127	1087	1238
**90%**	8759	2326	3.766	405	1023	1303
**95%**	9498	2326	4.083	479	977	1349

**Table 2:** Sequence comparison methods and parameters

**Method**	**Abbreviation**	**Version**	**Matrix**	**Gap open penalty**	**Gap extension penalty**	**Number of randomizations**
**Paracel SW e-value**	pc e	-	BLOSUM62	3*IS *	0.3*IS *	0
**Biofacet SW Z-score**	bf z	2.9.6	BLOSUM62	12	1	100
**NCBI BLAST e-value**	bl e	2.2.9	BLOSUM62	12	1	0
**FASTA e-value**	fa e	3.4t24	BLOSUM62	12	1	0
**SSEARCH e-value**	ss e	3.4t24	BLOSUM62	12	1	0
**ParAlign SW e-value**	pa e	4.0.0	BLOSUM62	12	1	0

* IS = average matrix identity score

### Receiver operating characteristic

**Figure 1:**
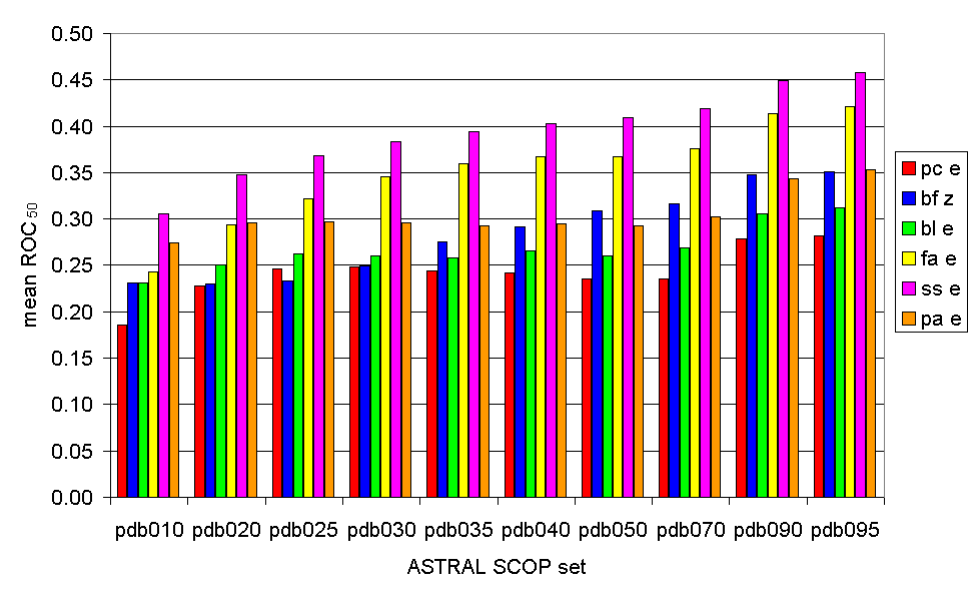
**The mean receiver operating characteristic scores for ten different ASTRAL SCOP sets**. The maximal structural identity percentage of each set increases from the left to the right, from 10% to 95%. Red bars: mean ROC50 scores calculated using the Paracel Smith-Waterman algorithm. Blue bars: mean ROC_50 _scores calculated using the Biofacet Smith-Waterman algorithm with Z-score statistics. Green bars: mean ROC_50 _scores calculated using the BLAST algorithm. Yellow bars: mean ROC_50 _scores calculated using the FASTA algorithm. Purple bars: mean ROC_50 _scores calculated using the SSEARCH algorithm. Orange bars: mean ROC_50 _scores calculated using the ParAlign Smith-Waterman algorithm.

The mean ROC_50 _scores increase if more structurally identical proteins are included, for both the e-value and the Z-score measurements (Fig. 1). The ROC_50 _scores of the PDB010 set show a large difference between the several Smith-Waterman implementations: 0.19 for Paracel, 0.23 for Biofacet (with Z-score), 0.27 for ParAlign and 0.31 for SSEARCH. The advantage of ParAlign over Biofacet decreases with increasing inclusiveness of the ASTRAL SCOP set that is used. The ROC_50 _scores of the PDB095 set are 0.28 for Paracel, 0.35 for both ParAlign and Biofacet (with Z-score) and 0.46 for SSEARCH. SSEARCH scores best of all studied methods, regardless of which ASTRAL SCOP set is used. The reference methods FASTA and BLAST give quite different results: FASTA is a good second and BLAST has scores similar to Paracel and Biofacet.

### Coverage versus error

**Figure 2:**
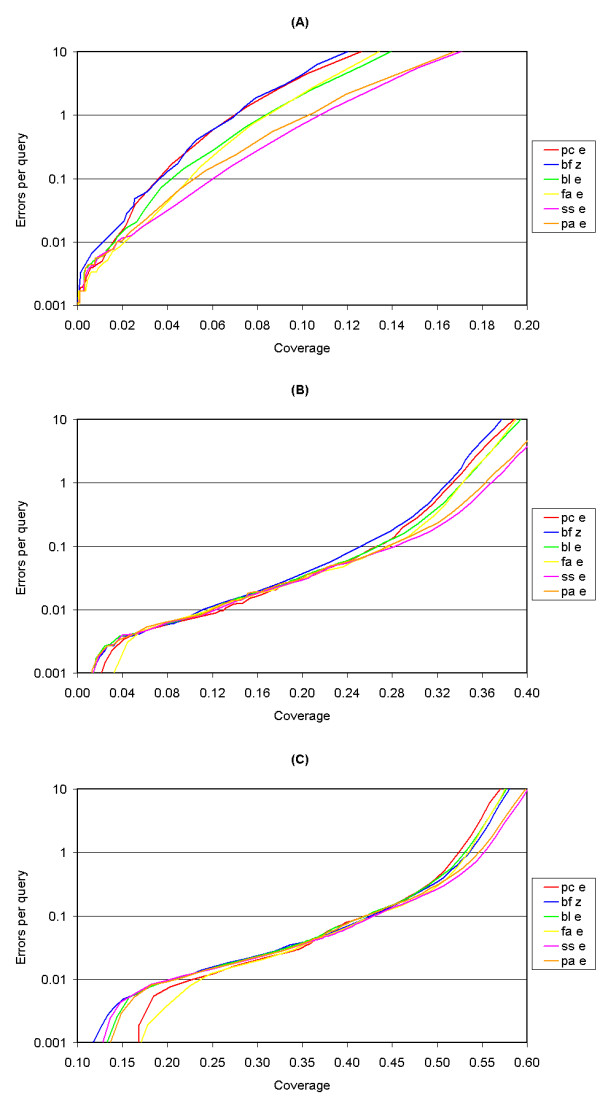
**(A) Coverage versus error plot for the ASTRAL SCOP PDB010 set. (B) Coverage versus error plot for the ASTRAL SCOP PDB035 set. (C) Coverage versus error plot for the ASTRAL SCOP PDB095 set**. Red line: calculated using the Paracel Smith-Waterman algorithm. Blue line: calculated using the Biofacet Smith-Waterman algorithm with Z-score statistics. Green line: calculated using the BLAST algorithm. Yellow line: calculated using the FASTA algorithm. Purple line: calculated using the SSEARCH algorithm. Orange line: calculated using the ParAlign Smith-Waterman algorithm.

This method differs from the ROC analysis on one crucial point: instead of looking at the first 100 hits, we varied the threshold at which a hit was seen as a positive. Hence the results are somewhat dissimilar: the differences between the several algorithms in the coverage versus error plots (Fig. 2) are not as obvious as they are in the ROC_50 _graph (Fig. 1). Figure 2A shows the coverage versus error plot for the smallest ASTRAL SCOP set (PDB010), figure 2B shows the plot for the largest ASTRAL SCOP set (PDB095) and figure 2C shows the plot for the intermediate set PDB035. An ideal algorithm would have a very high coverage but not many errors per query, which places it in the lower right corner of the graph. SSEARCH has the best scores when using the PDB010 set, followed by ParAlign and FASTA, with the latter scoring best in the lowest-coverage range (<0.02). Biofacet with Z-score has the lowest scores. The PDB095 plot shows some differences between the low-coverage range (<0.25) and the high-coverage range (>0.50). In the low coverage range, FASTA and Paracel have the highest scores, whereas SSEARCH and ParAlign have the highest scores in the low-coverage range. It should be noted that the high-coverage range might statistically be more reliable because of the larger number of hits. The PDB035 set gives similar results.

### Average precision

**Figure 3:**
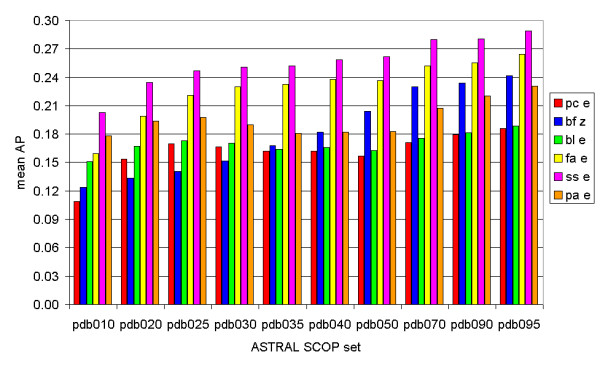
**The average precision values for ten different ASTRAL SCOP sets**. The maximal structural identity percentage of each set increases from the left to the right, from 10% to 95%. Red bars: mean AP values calculated using the Paracel Smith-Waterman algorithm. Blue bars: mean AP values calculated using the Biofacet Smith-Waterman algorithm with Z-score statistics. Green bars: mean AP values calculated using the BLAST algorithm. Yellow bars: mean AP values calculated using the FASTA algorithm. Purple bars: mean AP values calculated using the SSEARCH algorithm. Orange bars: mean AP values calculated using the ParAlign Smith-Waterman algorithm.

The average precision graph (Fig. 3) shows some minor differences from the ROC_50 _graph (Fig. 1): for the PDB020, PDB025 and PDB030 set, Paracel (e-value) scores better than Biofacet (Z-score). However, the advantage of the Biofacet Smith-Waterman with Z-score increases from that point on (PDB035, Paracel: 0.16, Biofacet: 0.17) to the right side (PDB095, Paracel: 0.19, Biofacet: 0.24). The Z-score seems to score better when more similar proteins are compared. Once more, SSEARCH has the highest scores for all structural identity percentages, with FASTA as the second best.

### Case studies

We included two examples of our statistical analysis, which show how the ROC and mean AP measures differ from each other and how results can be different for each studied protein. We choose two well-studied proteins: enoyl-ACP reductase and the progesterone receptor, the first from a prokaryote (*E. coli*) and the second from a eukaryote (*H. sapiens*). Both case studies were done using the PDB095 set, which is the most complete ASTRAL SCOP PDB set used in our study.

### Bacterial enoyl-ACP reductase

Table 3 shows the results of our analysis of the ASTRAL SCOP entry of *E. coli *enoyl-ACP reductase chain A, d1qg6a_, using the PDB095 set. One way of testing the reliability of a sequence comparison method is by looking at the first false positive (FFP) in the list of top 100 hits (Table S.1 [see Additional file 1]). The c.2.1.2 structural family has 46 members within the PDB095 set, so the perfect sequence comparison algorithm would return its first false positive at the 46^th ^hit (the hit containing the query protein is discarded). For the Paracel Smith-Waterman implementation, this is already the twenty-first hit. Four algorithms score best with the first false positive at 24^th ^place. A second testing method is counting the total number of true positives (NTP), of which the perfect algorithm would return all 45. BLAST has the highest score here: 27 out of the top 100 hits are true positives. FASTA and Paracel are at the second place with 25 true positives. Biofacet has the lowest score: only 23 true positives. Note that differences are very small, which is a reason to look at the ROC and mean AP scores. FASTA and SSEARCH have both the highest ROC_50 _scores and the highest mean APs. ParAlign and BLAST are third and fourth, followed by Paracel and Biofacet. The ROC and mean AP scores give a clearer view of the differences between the algorithms than the FFP or NTP scores, because they take into account the ranking of all hits instead of just the first false positive or just the true positives.

**Table 3:** Scores for bacterial enoyl-ACP reductase

	**pc e**	**bf z**	**bl e**	**fa e**	**ss e**	**pa e**
**ROC score**	0.156	0.124	0.250	0.367	0.338	0.229
**MAP score**	0.212	0.161	0.264	0.374	0.343	0.234
**First False Polsitive (FFP)**	21	24	24	22	24	24
**Number of True Positives (NTP)**	25	23	27	25	24	24

### Human progesterone receptor

Table 4 shows our analysis of ASTRAL SCOP entry d1a28a_, using again the PDB095 set. The structural family a.123.1.1 has 29 members, so the perfect algorithm should have the first false positive at the 29^th ^hit. Surprisingly, BLAST scores best here with its first false positive at the 25^th ^hit (Table S.2 [see Additional file 1]), although the differences are quite small. BLAST is, together with Biofacet, the only algorithm that does not have all the 28 family members of d1a28a_ in its top 100 list; d1n83a_ is missing here. The ROC_50 _and mean AP analysis of d1a28a_ shows again that SSEARCH and FASTA give the best results. Paracel and Biofacet have the lowest scores once more. The differences are not large enough to put any definite conclusions to the results of this example, but by combining all ROC and mean AP scores for all ASTRAL SCOP entries, we created a reliable comparison between all sequence comparison methods.

**Table 4:** Scores for human progesterone receptor

	**pc e**	**bf z**	**bl e**	**fa e**	**ss e**	**pa e**
**ROC score**	0.402	0.437	0.513	0.745	0.762	0.573
**MAP score**	0.504	0.503	0.548	0.727	0.745	0.586
**First False Positive (FFP)**	22	18	25	23	23	23
**Number of True Positives (NTP)**	28	27	27	28	28	28

### Timing

Table 5 shows the time that each of the six algorithms needs to perform an all-against-all sequence comparison of the ASTRAL SCOP PDB095 set. The BLAST algorithm is clearly the fastest, followed by the other heuristic algorithm FASTA. Of the Smith-Waterman algorithms, ParAlign is by far the fastest. The Biofacet algorithm needs much time to calculate 2 × 100 randomizations and is therefore the slowest sequence comparison algorithm.

**Table 5:** Times for all-against-all sequence comparisons of the ASTRAL SCOP PDB095 set.

**Method**	**Time**
**Paracel SW e-value**	3 hours *
**Biofacet SW Z-score**	multiple days
**NCBI BLAST e-value**	15 minutes
**FASTA e-value**	40 minutes
**SSEARCH e-value**	5 hours, 49 minutes
**ParAlign SW e-value**	47 minutes

* estimation because of unavailability Paracel system

## Discussion

**Figure 4:**
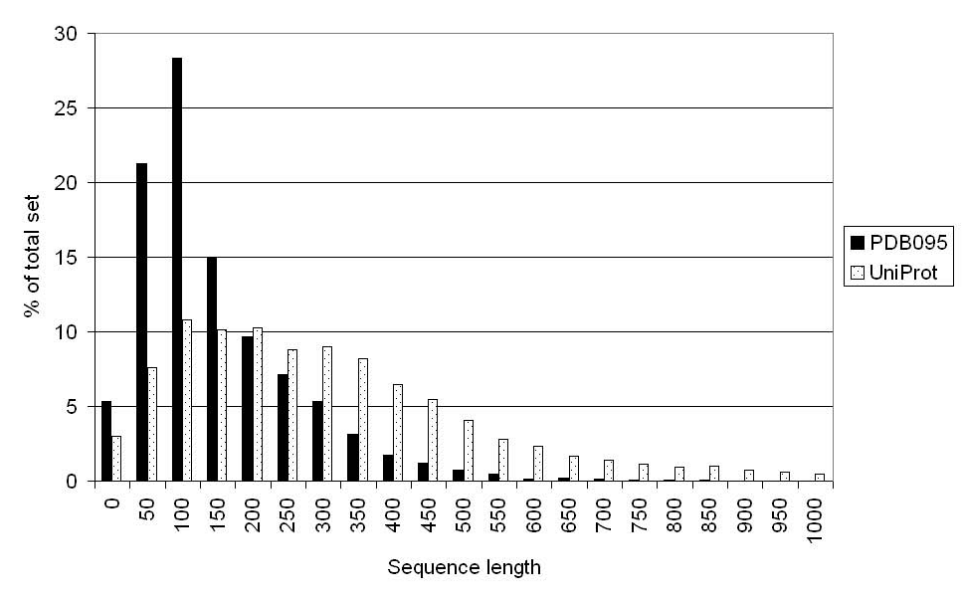
**Sequence length distribution between PDB095 and UniProt**. The sequence length increases from the left to the right. The vertical axis shows the number of proteins having that length, as a percentage of the total set. Black bars: PDB095 set. Dotted bars: UniProt set.

**Figure 5:**
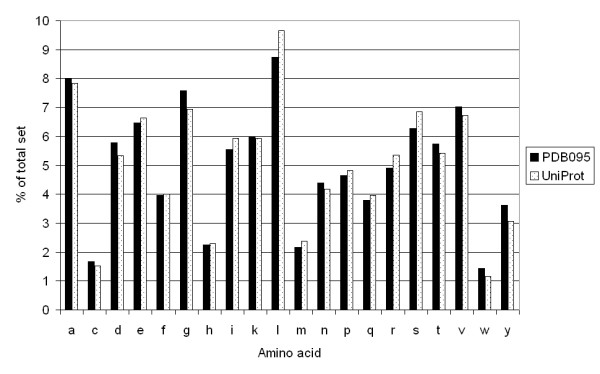
**Amino acid distribution between PDB095 and UniProt**. The 20 amino acids are displayed on the horizontal axis and their occurrence, as percentage of the total, is shown on the vertical axis. Black bars: PDB095 set. Dotted bars: UniProt set.

The theoretical advantage of the Z-score over the e-value appears to be rejected by our results. Our results show that the e-value calculation gives an advantage over the computationally intensive Z-score, at least when looking only at the results from the Smith-Waterman algorithm. Some caution should be taken however, drawing any definite conclusions. First, the Z-score was designed to make a distinction between significant hits and non-significant hits that have high SW scores. It might have an advantage over the e-value when applied to the top hits only, but might have less advantage for the hits with lower SW scores. This idea is supported by the fact that the Z-score is better at scoring high-similarity sequence pairs. This is also reflected in the different ROC and AP scores for the PDB010 set and the PDB095 set: the difference between Z-score and e-value increases when structurally more similar protein pairs are being included. Second, the Z-score can differ for each run, because of its different randomizations [17]. The standard deviation of the Z-score increases almost proportionally with the Z-score itself, i.e. for higher Z-scores the variance will be larger [16]. However, the Z-score increases its precision when more randomizations are calculated (2 × 100 in this study). Third, the PDB set is somewhat biased: it only contains crystallized proteins, and it contains no hypothetical proteins and membrane proteins. The crystallized proteins in the PDB are on average smaller than proteins included in large sequence databases such as the UniProt [26] database (Figure 4), whereas the amino acid distribution is approximately the same for these databases (Figure 5).

**Figure 6:**
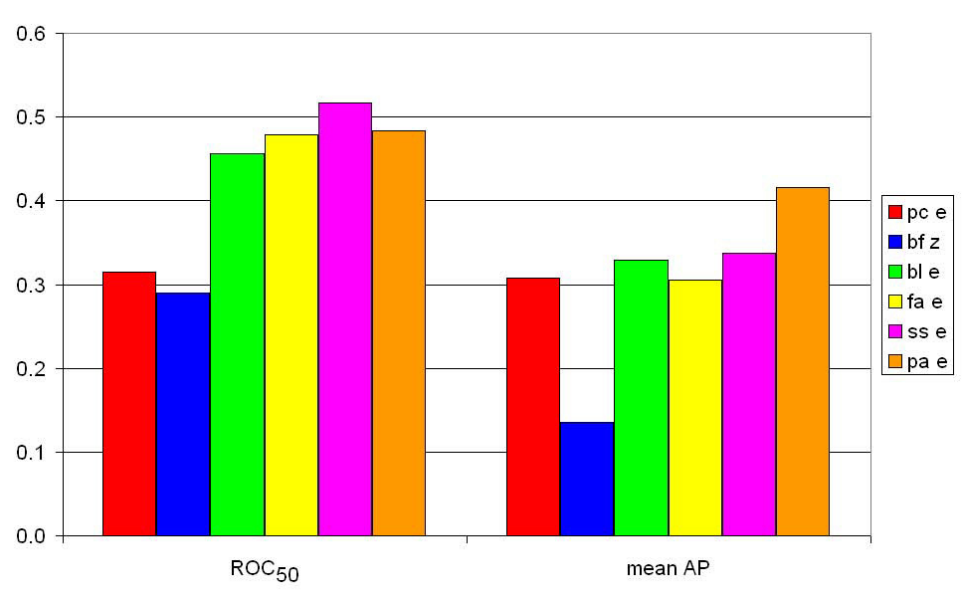
**ROC_50 _and mean AP values for proteins larger than 500 aa**. The ROC_50 _scores are shown at the left half, the mean AP values on the right half. Red bars: calculated using the Paracel Smith-Waterman algorithm. Blue bars: calculated using the Biofacet Smith-Waterman algorithm with Z-score statistics. Green bars: calculated using the BLAST algorithm. Yellow bars: calculated using the FASTA algorithm. Purple bars: calculated using the SSEARCH algorithm. Orange bars: calculated using the ParAlign Smith-Waterman algorithm.

**Figure 7:**
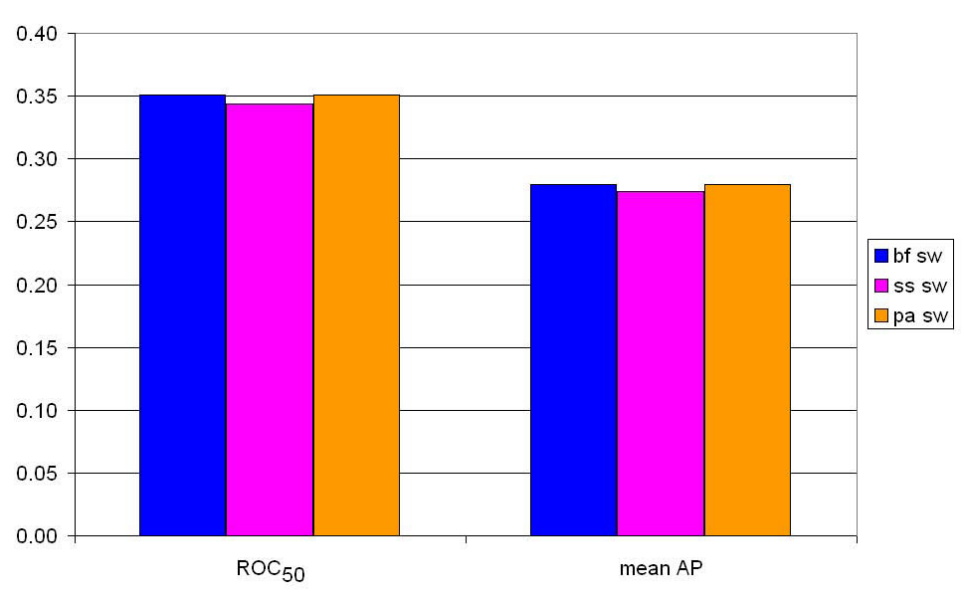
**ROC_50 _and mean AP values for the SW scores of three different SW algorithms**. The ROC_50 _scores are shown at the left half, the mean AP values on the right half. Blue bars: calculated using the Biofacet Smith-Waterman algorithm with Z-score statistics. Purple bars: calculated using the SSEARCH algorithm. Orange bars: calculated using the ParAlign Smith-Waterman algorithm.

Figure 6 shows that the bias in sequence length is not the reason for the difference in scores: if we only look at proteins with a sequence length of 500 or more, the scores are similar. Other studies have shown that FASTA performs better than BLAST [18,27], but these did not include several Smith-Waterman implementations. The SSEARCH algorithm, an implementation of Smith-Waterman, was analyzed in these studies, but this algorithm differs from other Smith-Waterman algorithms used in this study due to the use of length regression statistics [7,28]. A difference can also be found by comparing the SW scores of Biofacet, ParAlign and SSEARCH: Biofacet and ParAlign have the same SW scores, but the SSEARCH SW scores are different. We calculated the ROC_50 _and mean AP for these three SW scores and found that the SSEARCH SW scores gives slightly worse results than the other two SW scores (Figure 7). Another problem is that protein sequences within a certain ASTRAL SCOP family usually have equivalent lengths, since the ASTRAL SCOP database consists of protein domains and not of whole proteins. Results might vary when whole proteins, with different lengths, are studied. Unfortunately, the composition of the ASTRAL SCOP database does not allow us to confirm this statement.

Finally, we would like to stress that the results from the CVE analysis might be more reliable than those from the ROC and mean AP analyses. ROC and mean AP make use of a ranking system based on the e-value or Z-score, instead of looking at the e-value or Z-score directly. This means that in some cases, especially the smaller protein families, a large number of very low-scoring hits (e.g. e>100 or Z<3) is still used for the calculation of the scores. This is not the case for the CVE plots, because we varied the e-value and Z-score thresholds above which a hit is seen as a true positive, instead of relying on a ranking system. However, because the results from the CVE plots are similar to the results from the ROC and mean AP graphs, the use of a ranking system does not seem to give a large disadvantage.

## Conclusion

For a complete analysis we need a less biased database, having a wide range of proteins classified by structure similarity. Until such a database is available, it will be difficult to pinpoint the reasons for the different results between FASTA, BLAST and Smith-Waterman, and the theoretical advantages of the Z-score. Regardless of all these theoretical assumptions, the computational disadvantage of the Z-score is smaller for larger databases. Z-scores do not have to be recalculated when sequences are added to the database, in contrast to e-values, which are dependent on database size. For very large databases containing all-against-all comparisons, this is an important advantage of the Z-score. Although recalculating the e-values does not take much time when the alignments and SW scores are already available, this may cause a change in research results that were obtained earlier. Despite these considerations, we recommend using SSEARCH with e-value statistics for pairwise sequence comparisons.

## Methods

### Sequence comparisons

For the Smith-Waterman e-value calculation, the ASTRAL SCOP files were loaded onto the Paracel file system as protein databases and subsequently used as queries against these databases: the set with 10% maximal identity (PDB010) against itself, the set with 20% maximal identity (PDB020) against itself, etc. The matrix used for all sequence comparisons was the BLOSUM62 matrix [29]. This is the default scoring matrix for most alignment programs. For all sequence comparisons in this article, the gap open penalty was set to 12 and the gap extension penalty was set to 1. These are the averages of the default penalties over the six studied methods. Both the matrix and gap penalties used are suited for comparing protein sets with a broad spectrum of evolutionary distances, like the PDB set [30,31]. Per query sequence, the best 100 hits were kept [see section Data availability], discarding the match of each query sequence with itself.

### Receiver operating characteristic calculation

For each query, the 100 best hits were marked as true positives or false positives, i.e. the hit being in the same or in a different SCOP family than the query. For each of the first 50 false positives that were found, the number of true positives with a higher similarity score was calculated. The sum of all of these numbers was then divided by the number of false positives (50), and finally divided by the total number of possible true positives in the database (i.e. the total number of members in the SCOP family minus 1), giving an ROC_50 _score for each query sequence. The average of these ROC_50 _scores gives the final ROC score for that specific statistical value and that specific ASTRAL SCOP set. Mean ROC_50 _scores were calculated for all ten different ASTRAL SCOP sets.

### Coverage versus error calculation

Instead of taking the first 100 hits for each query, like in the ROC analysis, we varied the threshold at which a certain hit was seen as a positive. For the e-value analysis, we created a list of 49 thresholds in the range of 10^-50 ^to 100. For Z-score, we created a list of 58 thresholds in the range of 0 to 100. Then, for each threshold, two parameters were measured: the coverage and the errors per query (EPQ). The coverage is the number of true hits divided by the total number of sequence pairs that are in the same SCOP family, for that specific ASTRAL SCOP set. The EPQ is the number of false hits divided by the number of queries. We used the most inclusive ASTRAL SCOP set (PDB095), the least inclusive set (PDB010) and an intermediate set (PDB035) to create the coverage versus error plots.

### Average precision calculation

For the calculation of the average precision (AP), the 100 best hits per query were marked again as either true positives or false positives. Subsequently for each true positive found by the search algorithm, the true positive rank of this hit (i.e. the number of true positives with a higher score + 1) was divided by the positive rank (i.e. the number of hits with a higher score + 1). These numbers were all added up and then divided by the total number of hits (i.e. 100), giving one AP value per query. The mean AP is the average of all these APs. Mean APs were calculated for all ten different ASTRAL SCOP sets.

### Bacterial enoyl-ACP reductase

The ASTRAL SCOP entry for *E. coli *enoyl-ACP reductase chain A, d1qg6a_, was picked as an example for our methodology. The 100 best hits of this entry on the PDB095 set were calculated using each of the six algorithms and sorted by ascending e-value and descending Z-score. Then they were marked as either true positives or false positives, depending on if the hit was in the same structural family (c.2.1.2) or not. Furthermore, the ROC_50 _scores and mean APs were calculated.

### Human progesterone receptor

A second example is the analysis of d1a28a_, the *H. sapiens *progesterone receptor chain A. Once more, the 100 best hits of this entry on the PDB095 set were calculated using each of the six algorithms and sorted by ascending e-value and descending Z-score. These hits were marked as either true positives or false positives, depending on if the hit was in the same structural family (a.123.1.1) or not. Finally, the mean AP and ROC_50 _scores were calculated.

### Timing

We measured the speed of the sequence comparison algorithms, by doing an all-against-all comparison of the ASTRAL SCOP PDB095 set and using the 'time' command provided by UNIX. All calculations were performed on the same machine, except for the Paracel calculation which could only be performed on the Paracel machine. The Paracel calculation time had to be estimated because of the unaivailability of the Paracel machine at the time of performing this analysis.

## Data availability

All raw sequence comparison output files (containing the top 100 hits per query sequence) are available through our website [32]. The top 100 hits for the two case studies of the bacterial enoyl-ACP reductase (i.e. Table S.1) and the human progesterone receptor (i.e. Table S.2) can be found in the additional files section [see Additional file 1].

## Abbreviations

AP Average Precision

bf z Biofacet (Z-score)

BLAST Basic Local Alignment Search Tool

bl e BLAST (e-value)

BLOSUM BLOcks SUbstitution Matrix

CluSTr Clusters of SWISS-PROT and TrEMBL

CVE Coverage Versus Error

EPQ Errors Per Query

fa e FASTA (e-value)

FFP First False Positive

NTP Number of True Positives

pa e ParAlign (e-value)

pc e Paracel (e-value)

PDB Protein Data Bank

ROC Receiver Operating Characteristic

SCOP Structural Classification Of Proteins

ss e SSEARCH (e-value)

SW Smith-Waterman

## Authors' contributions

TH participated in the design of the study, carried out the calculations and statistical analysis and drafted the manuscript

JdV participated in the design of the study

JL gave some technical and scientific advice and helped to draft the manuscript

PG participated in the design and coordination of the study and helped to draft the manuscript

All authors read and approved the final manuscript

## Supplementary Material

Additional File 1 Supplementary tables: **Table S.1**. Top 100 hits of bacterial enoyl-ACP reductase. **Table S.2**. Top 100 hits of human progesterone receptor. Click here for file
