# Supplementary material for: Testing statistical significance scores of sequence comparison methods with structure similarity
Source: BMC Bioinformatics. 2006 Oct 12;7:444. doi: 10.1186/1471-2105-7-444 (PMC1618413; doi:10.1186/1471-2105-7-444)
Supplement: Additional File 1 — Supplementary tables: Table S.1. Top 100 hits of bacterial enoyl-ACP reductase. Table S.2. Top 100 hits of human progesterone receptor. [file 1471-2105-7-444-S1.doc]

**Table S.1.** Top 100 hits of bacterial enoyl-ACP reductase

| query | d1qg6a_ |  |  |  |  |  |  |  |  |  |  |  |
| --- | --- | --- | --- | --- | --- | --- | --- | --- | --- | --- | --- | --- |
| hit # | pc e |  | bf z |  | bl e |  | fa e |  | ss e |  | pa e |  |
| 1 | d1eno__ | 7.0E-25 | d1eny__ | 54.822 | d1eny__ | 2.0E-27 | d1eny__ | 1.3E-24 | d1eny__ | 1.7E-28 | d1eny__ | 1.0E-26 |
| 2 | d1eny__ | 2.0E-24 | d1eno__ | 48.901 | d1eno__ | 6.0E-27 | d1eno__ | 8.3E-24 | d1eno__ | 1.1E-27 | d1eno__ | 3.0E-26 |
| 3 | g1nhd.1 | 6.0E-19 | g1nhd.1 | 41.741 | g1nhd.1 | 3.0E-21 | g1nhd.1 | 2.7E-19 | g1nhd.1 | 1.4E-21 | g1nhd.1 | 1.0E-20 |
| 4 | d1fmca_ | 5.0E-14 | d1fmca_ | 37.042 | d1fmca_ | 4.0E-18 | d1fmca_ | 3.0E-16 | d1fmca_ | 6.4E-19 | d1fmca_ | 1.0E-17 |
| 5 | d1cyda_ | 6.0E-09 | d1ja9a_ | 27.808 | d1cyda_ | 2.0E-11 | d1cyda_ | 5.6E-10 | d1ja9a_ | 2.6E-13 | d1ja9a_ | 6.0E-12 |
| 6 | d1i01a_ | 4.0E-07 | d1cyda_ | 25.471 | d1g0oa_ | 2.0E-11 | d1ae1a_ | 2.7E-09 | d1cyda_ | 2.4E-12 | d1cyda_ | 5.0E-11 |
| 7 | d1ja9a_ | 4.0E-07 | d1g0oa_ | 25.324 | d1i01a_ | 2.0E-11 | d1iy8a_ | 2.7E-09 | d1g0oa_ | 4.9E-12 | d1i01a_ | 7.0E-11 |
| 8 | d1g0oa_ | 5.0E-06 | d1i01a_ | 22.056 | d1ja9a_ | 7.0E-11 | d1hxha_ | 4.4E-09 | d1i01a_ | 5.6E-12 | d1g0oa_ | 8.0E-11 |
| 9 | d1iy8a_ | 1.0E-05 | d1ae1a_ | 21.330 | d1iy8a_ | 1.0E-09 | d1i01a_ | 1.2E-08 | d1ae1a_ | 2.6E-10 | d1iy8a_ | 4.0E-09 |
| 10 | d1hxha_ | 9.0E-05 | d1hxha_ | 20.759 | d1ae1a_ | 2.0E-09 | d1ja9a_ | 1.2E-07 | d1iy8a_ | 2.6E-10 | d1ae1a_ | 5.0E-09 |
| 11 | d1ae1a_ | 2.0E-04 | d1iy8a_ | 20.431 | d1hxha_ | 3.0E-09 | d2ae2a_ | 1.6E-06 | d1hxha_ | 4.4E-10 | d1hxha_ | 9.0E-09 |
| 12 | d1fjha_ | 3.0E-04 | d2ae2a_ | 18.398 | d2ae2a_ | 1.0E-06 | d1gcoa_ | 5.6E-06 | d2ae2a_ | 3.4E-07 | d2ae2a_ | 4.0E-06 |
| 13 | d1gcoa_ | 6.0E-04 | d1gcoa_ | 16.536 | d1h5qa_ | 2.0E-06 | d1fjha_ | 2.5E-05 | d1h5qa_ | 4.5E-07 | d1gcoa_ | 6.0E-06 |
| 14 | d2ae2a_ | 0.001 | d1h5qa_ | 15.242 | d1fjha_ | 4.0E-06 | d1e7wa_ | 1.0E-04 | d1gcoa_ | 1.4E-06 | d1h5qa_ | 8.0E-06 |
| 15 | d1e7wa_ | 0.004 | d1e7wa_ | 14.976 | d1hdca_ | 6.0E-06 | d1h5qa_ | 0.0002 | d1hdca_ | 1.8E-06 | d1fjha_ | 1.0E-05 |
| 16 | d1h5qa_ | 0.027 | d1fjha_ | 14.394 | d1gcoa_ | 1.0E-05 | d1nffa_ | 0.00024 | d1e7wa_ | 2.8E-06 | d1hdca_ | 2.0E-05 |
| 17 | d1hdca_ | 0.14 | d1hdca_ | 14.390 | d1e7wa_ | 2.0E-05 | d1hdca_ | 0.0019 | d1fjha_ | 7.8E-06 | d1e7wa_ | 8.0E-05 |
| 18 | d1nffa_ | 0.24 | d1nffa_ | 13.520 | d1nffa_ | 3.0E-04 | d1jtva_ | 0.013 | d1nffa_ | 9.7E-05 | d1nffa_ | 8.0E-04 |
| 19 | d1nxqa_ | 4.8 | d1nxqa_ | 11.068 | d1gega_ | 0.001 | d1bdb__ | 0.093 | d1gega_ | 0.00057 | d1gega_ | 0.004 |
| 20 | d1gega_ | 8.2 | d1gega_ | 10.494 | d1edoa_ | 0.007 | d1g0oa_ | 0.33 | d1edoa_ | 0.00096 | d1nxqa_ | 0.006 |
| 21 | d1jiha_ | 14 | d1jtva_ | 8.773 | d1bdb__ | 0.021 | d1gega_ | 0.85 | d1bdb__ | 0.0011 | d1edoa_ | 0.018 |
| 22 | d1jtva_ | 24 | d1edoa_ | 8.554 | d1jtva_ | 0.021 | d1jiha_ | 2.7 | d1nxqa_ | 0.0013 | d1bdb__ | 0.065 |
| 23 | d4ubpc2 | 54 | d1bdb__ | 7.858 | d1nxqa_ | 0.27 | d1rpja_ | 4.4 | d1jtva_ | 0.0087 | d1jtva_ | 0.067 |
| 24 | d1e93a_ | 71 | d1biha2 | 6.012 | d4ubpc2 | 1.5 | d1d4ca3 | 4.9 | d4ubpc2 | 4.1 | d1jiha_ | 3.6 |
| 25 | d2bbkh_ | 71 | d1jiha_ | 5.916 | d1e93a_ | 3.5 | d1biha2 | 8.2 | d1jiha_ | 4.3 | d4ubpc2 | 6.4 |
| 26 | d2mpra_ | 93 | d1iuha_ | 5.845 | d2bbkh_ | 3.5 | d1pprm1 | 8.7 | d2ceva_ | 5.1 | d2ceva_ | 8.4 |
| 27 | g1nme.1 | 122 | g1qtn.1 | 5.750 | d1rpja_ | 4.6 | d2bbkh_ | 8.9 | d1d4ca3 | 6.4 | d1d4ca3 | 8.7 |
| 28 | d1eexa_ | 160 | d2mpra_ | 5.511 | d2mpra_ | 4.6 | d1ngr__ | 11 | d1rpja_ | 6.5 | d1biha2 | 11 |
| 29 | d1biha2 | 209 | g1nme.1 | 5.457 | d1d4ca3 | 6.1 | d1e93a_ | 12 | d1biha2 | 11 | d2bbkh_ | 13 |
| 30 | d1edoa_ | 209 | d1ivla_ | 5.369 | d1gu6a_ | 6.1 | d1izda_ | 13 | d1pprm1 | 12 | d1rpja_ | 14 |
| 31 | g1mtp.1 | 209 | d1e6oh1 | 5.228 | d2ceva_ | 6.1 | d1p5vb_ | 13 | d1bm3l1 | 13 | g1nme.1 | 15 |
| 32 | d1a4ya_ | 274 | d1e93a_ | 5.218 | g1nme.1 | 6.1 | d1fjgk_ | 15 | d1ngr__ | 13 | d1pprm1 | 16 |
| 33 | d1d4ca3 | 274 | d4ubpc2 | 5.134 | d1dpja_ | 8.1 | d2b3ia_ | 15 | d1iuha_ | 15 | d1bm3l1 | 18 |
| 34 | d1itwa_ | 274 | d1gu6a_ | 5.063 | d1itwa_ | 8.1 | d1htp__ | 16 | d2bbkh_ | 15 | d1e93a_ | 18 |
| 35 | d1izda_ | 274 | g1mtp.1 | 4.982 | d1biha2 | 11 | d1qnia1 | 16 | g1mtp.1 | 15 | g1mtp.1 | 18 |
| 36 | d1p5vb_ | 274 | d1yejh1 | 4.907 | d1ce2a2 | 11 | d1bfs__ | 17 | d1yejh1 | 16 | d1p5vb_ | 19 |
| 37 | g1qtn.1 | 274 | d1ngr__ | 4.784 | d1eq2a_ | 11 | d1dqea_ | 17 | g1nme.1 | 16 | d1iuha_ | 20 |
| 38 | d1dpja_ | 358 | d2bbkh_ | 4.746 | d1iuha_ | 11 | d1eq2a_ | 17 | d1ivla_ | 18 | d2mpra_ | 21 |
| 39 | d1eq2a_ | 358 | d1mlba1 | 4.724 | d1pprm1 | 11 | d1e6oh1 | 19 | d1p5vb_ | 18 | d1htp__ | 23 |
| 40 | d1f1ja_ | 358 | d2ceva_ | 4.656 | d1a4ya_ | 14 | d1e0ca2 | 21 | d1jglh1 | 20 | d1ivla_ | 24 |
| 41 | d1gu6a_ | 358 | d1bm3l1 | 4.632 | d1jala1 | 14 | d1hzzc_ | 21 | d1fjgk_ | 21 | d1ngr__ | 24 |
| 42 | d1htp__ | 358 | d1qnia1 | 4.626 | d1p5vb_ | 14 | d1gsoa1 | 26 | d2b3ia_ | 21 | d1jglh1 | 27 |
| 43 | d1m2oa3 | 358 | d1jglh1 | 4.528 | d1psoe_ | 14 | d1oy1a_ | 26 | d1e93a_ | 22 | d1e6oh1 | 28 |
| 44 | d1e5xa_ | 469 | d1m2oa3 | 4.501 | g1qtn.1 | 14 | d1f1ja_ | 29 | d1htp__ | 23 | d1izda_ | 28 |
| 45 | d1e6oh1 | 469 | d1hssa_ | 4.495 | d1bm3l1 | 19 | d1fx3a_ | 29 | d1qnia1 | 23 | d2b3ia_ | 28 |
| 46 | d1iuha_ | 469 | d1gpol1 | 4.466 | d1f1ja_ | 19 | d1itwa_ | 29 | d1ycsb1 | 23 | d1dpja_ | 29 |
| 47 | d1jyea_ | 469 | d1p5vb_ | 4.455 | d1htp__ | 19 | d1m2oa3 | 32 | d1bfs__ | 24 | d1gu6a_ | 30 |
| 48 | d1tlfa_ | 469 | d1mixa2 | 4.388 | d1k7ka_ | 19 | d1mixa2 | 32 | d1dpja_ | 24 | d1qnia1 | 31 |
| 49 | d1bm3l1 | 614 | d1d4ca3 | 4.356 | d1m2oa3 | 19 | d1liua2 | 33 | d1izda_ | 24 | d1ycsb1 | 31 |
| 50 | d1bxna1 | 614 | d1fh5l1 | 4.314 | d1opoa_ | 19 | d1qs0b2 | 35 | d1mlba1 | 24 | d1bfs__ | 32 |
| 51 | d1c8da_ | 614 | d1kb2a_ | 4.292 | d1a4sa_ | 25 | d1e1oa1 | 37 | d1dqea_ | 25 | d1mlba1 | 32 |
| 52 | d1hssa_ | 614 | d1irza_ | 4.261 | d1dqea_ | 25 | d1m45a_ | 38 | d2mpra_ | 25 | d1dqea_ | 33 |
| 53 | d1psoe_ | 614 | d3tss_2 | 4.245 | d1e5xa_ | 25 | d1bj7__ | 39 | d1ce2a2 | 27 | d1eq2a_ | 35 |
| 54 | d1rpja_ | 614 | d1dpja_ | 4.239 | d1e6oh1 | 25 | d1kb2a_ | 39 | d1gu6a_ | 27 | g1qtn.1 | 36 |
| 55 | d1ub0a_ | 614 | d1ce2a2 | 4.237 | d1hzzc_ | 25 | d1n5ua3 | 39 | d1leha1 | 27 | d1fjgk_ | 37 |
| 56 | d4sbva_ | 614 | d1e0ca2 | 4.234 | d1ivla_ | 25 | d4sbva_ | 39 | d1e6oh1 | 28 | d1hssa_ | 37 |
| 57 | d4sbvc_ | 614 | d1dqea_ | 4.221 | d1jglh1 | 25 | d1jyea_ | 41 | d1eq2a_ | 30 | d1yejh1 | 38 |
| 58 | d1b26a1 | 803 | d1kb4b_ | 4.185 | d1jiha_ | 25 | d1yejh1 | 41 | d1fh5l1 | 32 | d1ce2a2 | 41 |
| 59 | d1bj7__ | 803 | d1iyca_ | 4.174 | d1jyea_ | 25 | d2tpt_1 | 41 | d1k4cb1 | 32 | d1k7ka_ | 41 |
| 60 | d1ce2a2 | 803 | d1f1ja_ | 4.155 | d1l5sa_ | 25 | d1cr1a_ | 42 | d1k7ka_ | 32 | d1fh5l1 | 42 |
| 61 | d1dqea_ | 803 | d1rpja_ | 4.139 | d1leha1 | 25 | d1ixra1 | 42 | d1psoe_ | 32 | d1jala1 | 42 |
| 62 | d1e1oa1 | 803 | d1htp__ | 4.129 | d1oy1a_ | 25 | d1jnya1 | 42 | d1e0ca2 | 33 | d1k4cb1 | 42 |
| 63 | d1gz6a_ | 803 | d1sgpi_ | 4.108 | d1qnia1 | 25 | d4sbvc_ | 43 | d1opoa_ | 33 | d1hzzc_ | 44 |
| 64 | d1hzzc_ | 803 | d1ycsb1 | 4.081 | d1tlfa_ | 25 | d1a4sa_ | 44 | d1hzzc_ | 34 | d1leha1 | 45 |
| 65 | d1ivla_ | 803 | d1opoa_ | 4.073 | d1ycsb1 | 25 | d1g4us1 | 44 | d1oy1a_ | 34 | d1f1ja_ | 48 |
| 66 | d1jala1 | 803 | d1af7_1 | 4.061 | d1cr1a_ | 33 | d1kb4b_ | 44 | d1jala1 | 35 | d1eexa_ | 50 |
| 67 | d1jglh1 | 803 | d1igyb1 | 4.056 | d1g72a_ | 33 | d1tlfa_ | 44 | d1n1ta1 | 35 | d1psoe_ | 50 |
| 68 | d1jqia1 | 803 | d1bz7a1 | 4.054 | d1gy8a_ | 33 | d1af7_1 | 46 | d1hssa_ | 37 | d1gsoa1 | 51 |
| 69 | d1k77a_ | 803 | d1gsoa1 | 4.049 | d1h3fa1 | 33 | d1itza3 | 46 | d1gsoa1 | 38 | d1opoa_ | 53 |
| 70 | d1l1la_ | 803 | d2b3ia_ | 4.017 | d1hssa_ | 33 | d1j83a_ | 46 | g1qtn.1 | 39 | d1m2oa3 | 54 |
| 71 | d1m45a_ | 803 | d1h32a2 | 4.013 | d1i6vd_ | 33 | d1irza_ | 48 | d1j1ol_ | 42 | d1bz7a1 | 56 |
| 72 | d1ooea_ | 803 | d1e1oa1 | 3.989 | d1mlba1 | 33 | d1dk7a_ | 49 | d1gpol1 | 45 | d1j1ol_ | 56 |
| 73 | d1a8i__ | 1051 | d2sob__ | 3.945 | d1ngr__ | 33 | d1gpl_1 | 49 | d1fx3a_ | 46 | d1e0ca2 | 57 |
| 74 | d1bz7a1 | 1051 | d1psoe_ | 3.937 | d1ngva_ | 33 | d1myt__ | 49 | d1mixa2 | 46 | d1oy1a_ | 57 |
| 75 | d1e9yb2 | 1051 | d1gpl_1 | 3.928 | d1ub0a_ | 33 | d1igyb1 | 50 | d1dova_ | 47 | d1kb2a_ | 58 |
| 76 | d1f76a_ | 1051 | d1jj2p_ | 3.904 | d1yejh1 | 33 | d1ub0a_ | 50 | d1fc6a4 | 50 | d1gpol1 | 59 |
| 77 | d1gece_ | 1051 | d1b12a_ | 3.902 | d4sbva_ | 33 | d1fbih1 | 53 | d1f1ja_ | 52 | d1dova_ | 60 |
| 78 | d1h16a_ | 1051 | d1a4ya_ | 3.865 | d4sbvc_ | 33 | d1cpq__ | 56 | d1ag8a_ | 55 | d1e1oa1 | 61 |
| 79 | d1i6vd_ | 1051 | d1jala1 | 3.836 | d1ag8a_ | 44 | d1gy8a_ | 56 | d1dowa_ | 55 | d1fx3a_ | 61 |
| 80 | d1itza3 | 1051 | d1jli__ | 3.829 | d1b26a1 | 44 | d1b26a1 | 59 | d1bz7a1 | 56 | d1mixa2 | 61 |
| 81 | d1jqoa_ | 1051 | d1jhja_ | 3.765 | d1bj7__ | 44 | d1lb3a_ | 59 | d1qs0b2 | 57 | d1m45a_ | 62 |
| 82 | d1k7ka_ | 1051 | d1k7ka_ | 3.764 | d1fh5l1 | 44 | d1ooea_ | 59 | d2tpt_1 | 58 | d1bj7__ | 64 |
| 83 | d1l5sa_ | 1051 | d1g4us1 | 3.741 | d1gz6a_ | 44 | d1ej8a_ | 60 | d1kb2a_ | 59 | d1irza_ | 64 |
| 84 | d1lb3a_ | 1051 | d1g1sa2 | 3.731 | d1jqia1 | 44 | d1kid__ | 63 | d1m2oa3 | 60 | d1jqia1 | 66 |
| 85 | d1llfa_ | 1051 | d1k77a_ | 3.727 | d1k4cb1 | 44 | d1ll7a1 | 63 | d1ixra1 | 61 | d1itwa_ | 67 |
| 86 | d1m6sa_ | 1051 | d1fjgk_ | 3.714 | d1l1la_ | 44 | d1n1qa_ | 64 | d1e1oa1 | 62 | d1kb4b_ | 67 |
| 87 | d1mlba1 | 1051 | d1pprm1 | 3.707 | d1liua2 | 44 | d1hv9a1 | 66 | d1eexa_ | 63 | d4sbva_ | 67 |
| 88 | d1myt__ | 1051 | d1igtb3 | 3.662 | d1m45a_ | 44 | d1kxqe_ | 67 | d1liua2 | 63 | d1af7_1 | 68 |
| 89 | d1n11a_ | 1051 | d1hxra_ | 3.662 | d1ogsa2 | 44 | d1ogsa2 | 67 | d1m45a_ | 63 | d1dowa_ | 70 |
| 90 | d1ohla_ | 1051 | d1bj7__ | 3.655 | d1udc__ | 44 | d1t7pa1 | 67 | d1jnya1 | 64 | d1a4ya_ | 72 |
| 91 | d1oy1a_ | 1051 | d1qs0b2 | 3.655 | d1a8i__ | 59 | d1e5xa_ | 69 | d1bj7__ | 66 | d1jyea_ | 72 |
| 92 | d1pprm1 | 1051 | d1qba_2 | 3.646 | d1b9ha_ | 59 | d1l5sa_ | 69 | d1jqia1 | 67 | d2tpt_1 | 73 |
| 93 | d1qhma_ | 1051 | d2ovo__ | 3.638 | d1e9yb2 | 59 | d1yedb1 | 69 | d1irza_ | 68 | d1qs0b2 | 74 |
| 94 | d2b3ia_ | 1051 | d1k4cb1 | 3.631 | d1ex9a_ | 59 | d1gece_ | 70 | d1kb4b_ | 68 | d1itza3 | 76 |
| 95 | d2ceva_ | 1051 | d1c8da_ | 3.630 | d1f0ka_ | 59 | d1jj2p_ | 70 | d1itwa_ | 69 | d4sbvc_ | 76 |
| 96 | d1fh5l1 | 1376 | d1ogsa2 | 3.626 | d1f76a_ | 59 | d1ioka3 | 72 | d1af7_1 | 70 | d1h32a2 | 78 |
| 97 | d1h32a2 | 1376 | d1h3ob_ | 3.589 | d1ih7a2 | 59 | d1jk9b1 | 73 | d1gjta_ | 70 | d1ixra1 | 78 |
| 98 | d1k4cb1 | 1376 | d1lpba2 | 3.575 | d1jqoa_ | 59 | d1gz6a_ | 75 | d1n5ua3 | 70 | d1gpl_1 | 79 |
| 99 | d1kb2a_ | 1376 | d1bfs__ | 3.566 | d1ll7a1 | 59 | d1kvna_ | 76 | d1dnpa2 | 71 | d1tlfa_ | 79 |
| 100 | d1kb4b_ | 1376 | d1m6ya2 | 3.546 | d1ohla_ | 59 | d1onwa1 | 77 | d4sbva_ | 71 | d1igyb1 | 81 |

**Table S.2.** Top 100 hits of the human progesterone receptor

| query | d1a28a_ |  |  |  |  |  |  |  |  |  |  |  |
| --- | --- | --- | --- | --- | --- | --- | --- | --- | --- | --- | --- | --- |
| hit # | pc e |  | bf z |  | bl e |  | fa e |  | ss e |  | pa e |  |
| 1 | d1gs4a_ | 3.0E-81 | d1gs4a_ | 150.516 | d1gs4a_ | 4.0E-85 | d1gs4a_ | 7.4E-81 | d1gs4a_ | 1.70E-87 | d1gs4a_ | 6.00E-86 |
| 2 | d1nhza_ | 1.0E-76 | d1nhza_ | 145.034 | d1nhza_ | 4.0E-80 | d1nhza_ | 1.3E-76 | d1nhza_ | 6.80E-83 | d1nhza_ | 3.00E-81 |
| 3 | d1l2ia_ | 2.0E-21 | d1l2ia_ | 48.250 | d1l2ia_ | 2.0E-21 | d1l2ia_ | 3.2E-22 | d1l2ia_ | 8.20E-24 | d1l2ia_ | 7.00E-23 |
| 4 | d1kv6a_ | 9.0E-21 | d1kv6a_ | 45.500 | d1kv6a_ | 1.0E-19 | d1kv6a_ | 2.0E-20 | d1kv6a_ | 7.10E-22 | d1kv6a_ | 3.00E-21 |
| 5 | d1qkna_ | 2.0E-18 | d1qkma_ | 41.212 | d1qkna_ | 3.0E-17 | d1qkna_ | 1.3E-19 | d1qkna_ | 5.70E-21 | d1qkna_ | 6.00E-20 |
| 6 | d1qkma_ | 2.0E-18 | d1qkna_ | 37.653 | d1qkma_ | 9.0E-17 | d1qkma_ | 3.9E-19 | d1qkma_ | 1.80E-20 | d1qkma_ | 2.00E-19 |
| 7 | d1g2na_ | 4.0E-10 | d1g2na_ | 31.943 | d1g2na_ | 7.0E-13 | d1g2na_ | 1.6E-10 | d1g2na_ | 2.40E-14 | d1g2na_ | 1.00E-13 |
| 8 | d1mzna_ | 5.0E-07 | d1mzna_ | 25.334 | d1pk5a_ | 4.0E-09 | d1mzna_ | 3.4E-09 | d1pk5a_ | 4.90E-10 | d1pk5a_ | 1.00E-09 |
| 9 | d1pk5a_ | 5.0E-07 | d1pk5a_ | 22.749 | d1mzna_ | 5.0E-08 | d1pk5a_ | 1.7E-08 | d1mzna_ | 1.10E-09 | d1mzna_ | 6.00E-09 |
| 10 | d1h9ua_ | 4.0E-06 | d1h9ua_ | 20.337 | d1h9ua_ | 8.0E-08 | d1h9ua_ | 2.7E-08 | d1h9ua_ | 1.00E-08 | d1h9ua_ | 4.00E-08 |
| 11 | d1m7wa_ | 1.0E-04 | d1m7wa_ | 17.551 | d1m7wa_ | 1.0E-07 | d1m7wa_ | 7.7E-08 | d1m7wa_ | 3.30E-08 | d1m7wa_ | 2.00E-07 |
| 12 | d1lv2a_ | 2.0E-04 | d1lv2a_ | 17.445 | d1lv2a_ | 4.0E-07 | d1lv2a_ | 6.4E-07 | d1lv2a_ | 3.30E-07 | d1lv2a_ | 1.00E-06 |
| 13 | d1hg4a_ | 3.0E-04 | d1hg4a_ | 17.303 | d1hg4a_ | 2.0E-05 | d1hg4a_ | 1.3E-05 | d1hg4a_ | 1.20E-06 | d1hg4a_ | 8.00E-06 |
| 14 | d1dkfb_ | 0.001 | d1fcya_ | 14.616 | d1dkfb_ | 2.0E-05 | d1dkfb_ | 2.0E-05 | d1dkfb_ | 1.40E-05 | d1dkfb_ | 4.00E-05 |
| 15 | d1fcya_ | 0.001 | d1dkfb_ | 14.365 | d1fcya_ | 2.0E-05 | d1fcya_ | 2.6E-05 | d1fcya_ | 1.90E-05 | d1fcya_ | 5.00E-05 |
| 16 | d2prga_ | 0.059 | d1ovla_ | 11.758 | d2prga_ | 0.002 | d2prga_ | 0.0012 | d1ovla_ | 0.00043 | d1ovla_ | 0.003 |
| 17 | d1i7ga_ | 0.18 | d1ovle_ | 11.274 | d1ovle_ | 0.002 | d1ovla_ | 0.0039 | d1ovle_ | 0.0005 | d1ovle_ | 0.004 |
| 18 | d1ie9a_ | 0.23 | d1e6ua_ | 11.172 | d1ovla_ | 0.002 | d1ovle_ | 0.0044 | d2prga_ | 0.0012 | d2prga_ | 0.007 |
| 19 | d1p8da_ | 0.69 | d1pdua_ | 9.622 | d1i7ga_ | 0.007 | d1pdua_ | 0.0065 | d1pdua_ | 0.0073 | d1i7ga_ | 0.02 |
| 20 | d1ovla_ | 1.2 | d1p8da_ | 9.427 | d1pdua_ | 0.016 | d1i7ga_ | 0.013 | d1i7ga_ | 0.016 | d1pdua_ | 0.022 |
| 21 | d1ovle_ | 1.2 | d1ie9a_ | 8.743 | d1p8da_ | 0.037 | d1ie9a_ | 0.016 | d1ie9a_ | 0.02 | d1ie9a_ | 0.025 |
| 22 | d1e6ua_ | 1.2 | d2prga_ | 8.582 | d1nava_ | 0.037 | d1p8da_ | 0.02 | d1p8da_ | 0.025 | d1p8da_ | 0.042 |
| 23 | d2gwxa_ | 1.6 | d1nava_ | 8.203 | d2gwxa_ | 0.065 | d1e6ua_ | 0.025 | d1e6ua_ | 0.034 | d1e6ua_ | 0.055 |
| 24 | d1pdua_ | 2 | d1i7ga_ | 7.988 | d1ie9a_ | 0.11 | d1nava_ | 0.036 | d1nava_ | 0.048 | d1nava_ | 0.11 |
| 25 | d1nava_ | 3.5 | d2gwxa_ | 7.717 | d1e6ua_ | 0.63 | d1m13a_ | 0.087 | d1m13a_ | 0.097 | d2gwxa_ | 0.19 |
| 26 | d1m13a_ | 14 | d1m13a_ | 6.219 | d1qjba_ | 1.1 | d2gwxa_ | 0.1 | d2gwxa_ | 0.15 | d1m13a_ | 0.48 |
| 27 | d1n46a_ | 40 | d1gz7a_ | 6.053 | d1n46a_ | 1.5 | d1qjba_ | 0.75 | d1k04a_ | 1.2 | d1qjba_ | 2.7 |
| 28 | d1gz7a_ | 40 | d1n46a_ | 5.841 | d1nowa1 | 2.6 | d1n46a_ | 1 | d1qjba_ | 1.3 | d1n46a_ | 3.9 |
| 29 | d1h1wa_ | 90 | d1b1ca_ | 5.758 | d1k04a_ | 3.4 | d1i5pa3 | 2.4 | d1n46a_ | 1.9 | d1k04a_ | 4.7 |
| 30 | d2oata_ | 90 | d1o6wa2 | 5.693 | d1i5pa3 | 4.5 | d1gz8a_ | 2.7 | d1ktma_ | 2.1 | d1ktma_ | 6 |
| 31 | d1qjba_ | 118 | d1qjba_ | 5.632 | d2oata_ | 4.5 | d1nowa1 | 4.1 | d1nowa1 | 2.8 | d1gz8a_ | 8.2 |
| 32 | d1b1ca_ | 154 | d1nowa1 | 5.466 | d1ktma_ | 6 | d1gz7a_ | 4.6 | d1k40a_ | 3.3 | d1k40a_ | 9.4 |
| 33 | d1ogae2 | 202 | d1ogae2 | 5.315 | d1b1ca_ | 8 | d1fhe_1 | 4.8 | d1i5pa3 | 4.7 | d1nowa1 | 9.8 |
| 34 | d1nowa1 | 202 | d1gz8a_ | 5.291 | d1ogae2 | 11 | d1b1ca_ | 5.9 | d1gz8a_ | 5.4 | d1gz7a_ | 11 |
| 35 | d1k4wa_ | 265 | d1k04a_ | 5.231 | d1k4wa_ | 14 | d1ogae2 | 5.9 | d1fhe_1 | 8.4 | d1i5pa3 | 13 |
| 36 | d1n83a_ | 265 | d1cpq__ | 5.209 | d1k40a_ | 14 | d2oata_ | 7.9 | d1ogae2 | 10 | d1ogae2 | 13 |
| 37 | d1ja1a2 | 347 | d5pnt__ | 5.070 | d1ia9a_ | 19 | d1he1a_ | 8.2 | d1b1ca_ | 11 | d1b1ca_ | 13 |
| 38 | d1f75a_ | 347 | d1ja1a2 | 4.949 | d1gyta2 | 19 | d1a04a2 | 8.3 | d1gz7a_ | 11 | d1h1wa_ | 14 |
| 39 | d1oaa__ | 347 | d1h1wa_ | 4.932 | d1ja1a2 | 19 | d1k4wa_ | 8.4 | d1h1wa_ | 12 | d1o6wa2 | 15 |
| 40 | d1ia9a_ | 347 | d1ktma_ | 4.915 | d1oaa__ | 19 | d1fewa_ | 10 | d1he1a_ | 15 | d2oata_ | 20 |
| 41 | d1gz8a_ | 347 | d1bor__ | 4.869 | d1m13a_ | 19 | d1mo9a3 | 11 | d1a04a2 | 15 | d1he1a_ | 24 |
| 42 | d1gyta2 | 347 | d2oata_ | 4.841 | d1fewa_ | 19 | d1o6wa2 | 11 | d1o6wa2 | 16 | d1kfta_ | 29 |
| 43 | d1ktma_ | 454 | d1i5pa3 | 4.679 | d1gz7a_ | 25 | d1ezvb2 | 12 | d1k4wa_ | 18 | d1bor__ | 29 |
| 44 | d5pnt__ | 454 | d1iala_ | 4.617 | d1ekea_ | 25 | d1fmta2 | 12 | d2oata_ | 19 | d1fhe_1 | 31 |
| 45 | d1fmta2 | 454 | d1kfta_ | 4.599 | d5pnt__ | 25 | d1sssa1 | 12 | d1fewa_ | 20 | d1fewa_ | 32 |
| 46 | d1ljya1 | 454 | d1k40a_ | 4.580 | d1fhe_1 | 25 | d1ja1a2 | 14 | d1mo9a3 | 21 | d1a04a2 | 32 |
| 47 | d1fhe_1 | 594 | d1gyta2 | 4.403 | d1cm8a_ | 33 | d1n83a_ | 15 | d1sssa1 | 21 | d1sssa1 | 32 |
| 48 | d1fewa_ | 594 | d1he1a_ | 4.380 | d1ezvb2 | 33 | d1iala_ | 15 | d1ezvb2 | 23 | d1ja1a2 | 33 |
| 49 | d1rypk_ | 594 | d1vcpa_ | 4.336 | d1db3a_ | 33 | d1ekea_ | 17 | d1fmta2 | 25 | d1mo9a3 | 33 |
| 50 | d1n45a_ | 594 | d1kfwa2 | 4.318 | d1n7oa1 | 33 | d1oaa__ | 19 | d1xo1a1 | 26 | d1o6wa1 | 33 |
| 51 | d1g0ha_ | 594 | d1xbl__ | 4.291 | d1gx5a_ | 44 | d1ia9a_ | 21 | d1kfta_ | 28 | d1k4wa_ | 35 |
| 52 | d1pa2a_ | 594 | d1rro__ | 4.266 | d1ebfa2 | 44 | d1cm8a_ | 25 | d1bor__ | 28 | d1n83a_ | 37 |
| 53 | d1db3a_ | 594 | d1mo9a3 | 4.212 | d1js3a_ | 44 | d1gvna_ | 27 | d1ja1a2 | 28 | d5pnt__ | 38 |
| 54 | d1n7oa1 | 594 | d1a04a2 | 4.139 | d2phla1 | 44 | d1nboa2 | 28 | d5pnt__ | 32 | d1f75a_ | 44 |
| 55 | d1jjya_ | 594 | d1nboa2 | 4.070 | d1he1a_ | 44 | d1ljya1 | 28 | d1n83a_ | 32 | d1ezvb2 | 47 |
| 56 | d1n08a_ | 778 | d1ljya1 | 4.063 | d1bjna_ | 58 | d1o0sa2 | 28 | d1n3la_ | 35 | d1oaa__ | 50 |
| 57 | d1ebfa2 | 778 | d7gata_ | 4.040 | d1dqna_ | 58 | d1fo4a6 | 29 | d1ekea_ | 38 | d1fmta2 | 51 |
| 58 | d2phla1 | 778 | d1bola_ | 3.990 | d1j5sa_ | 58 | d1zpda2 | 32 | d1iala_ | 38 | d1iala_ | 52 |
| 59 | d1ekea_ | 778 | d1o6wa1 | 3.987 | d1o6wa2 | 58 | d1g8ia_ | 32 | d1f75a_ | 39 | d1xo1a1 | 53 |
| 60 | d1iz7a_ | 778 | d1kola2 | 3.986 | d1vcpa_ | 58 | d1ebfa2 | 32 | d1p16a1 | 39 | d1ia9a_ | 54 |
| 61 | d1hjxa1 | 778 | d1iz7a_ | 3.944 | d1cvua1 | 58 | d1kola2 | 33 | d1d4ca3 | 39 | d1gvna_ | 56 |
| 62 | d1ihua1 | 778 | d1e0la_ | 3.943 | d1xo1a1 | 58 | d1vcpa_ | 33 | d1i1ga2 | 43 | d1ekea_ | 57 |
| 63 | d1e9la1 | 778 | d1ezvb2 | 3.900 | d1dgsa1 | 58 | d2phla1 | 34 | d1gyta2 | 45 | d1gyta2 | 64 |
| 64 | d1js3a_ | 778 | d1f1ga_ | 3.887 | d1g8ia_ | 58 | d1h6gb1 | 34 | d1oaa__ | 45 | d1n08a_ | 65 |
| 65 | d1o6wa2 | 1019 | d1fhe_1 | 3.885 | d1h1wa_ | 77 | d1pcfa_ | 35 | d1gvna_ | 49 | d1rypk_ | 65 |
| 66 | d1k40a_ | 1019 | d1fewa_ | 3.867 | d1gz8a_ | 77 | d1mgta2 | 35 | d1ia9a_ | 49 | d1pcfa_ | 65 |
| 67 | d1a26_1 | 1019 | d1f75a_ | 3.864 | d1icra_ | 77 | d1ix9a1 | 35 | d1dgsa1 | 51 | d1n3la_ | 67 |
| 68 | d1he1a_ | 1019 | d1sssa1 | 3.792 | d1nboa2 | 77 | d1c16a2 | 37 | d1n08a_ | 55 | d1d9aa_ | 71 |
| 69 | d1mo9a3 | 1019 | d1ekea_ | 3.733 | d1mira_ | 77 | d1m6ba2 | 38 | d1rypk_ | 57 | d1n45a_ | 71 |
| 70 | d1k04a_ | 1019 | d1ebfa2 | 3.731 | d1m6sa_ | 77 | d1cpq__ | 38 | d1cvua1 | 59 | d1a26_1 | 73 |
| 71 | d1vcpa_ | 1019 | d1bjna_ | 3.708 | d3tata_ | 77 | d1h6ga1 | 39 | d1nboa2 | 59 | d1ljya1 | 76 |
| 72 | d1m6ba2 | 1019 | d1nkd__ | 3.644 | d1cjya2 | 77 | d1cs8a_ | 40 | d1pcfa_ | 60 | d1ix9a1 | 77 |
| 73 | d1llna_ | 1019 | d1n45a_ | 3.636 | d1zpda2 | 77 | d1itha_ | 41 | d1d9aa_ | 62 | d1xbl__ | 79 |
| 74 | d1o0sa2 | 1019 | d4aig__ | 3.635 | d1ile_3 | 77 | d1p4ma_ | 43 | d1a26_1 | 63 | d1vcpa_ | 82 |
| 75 | d1d8ia_ | 1019 | d1k4wa_ | 3.629 | d1fvpa_ | 77 | d1ig3a1 | 44 | d1n45a_ | 63 | d1ebfa2 | 82 |
| 76 | d1bjna_ | 1019 | d1b67a_ | 3.622 | d1itua_ | 77 | d1rmd_2 | 44 | d1dula_ | 64 | d1g0ha_ | 85 |
| 77 | d1iala_ | 1019 | d1d4ca3 | 3.617 | d2ebn__ | 77 | d1i9ba_ | 45 | d1cm8a_ | 64 | d1i1ga2 | 86 |
| 78 | d1bor__ | 1333 | d1zpda2 | 3.611 | d1df0a2 | 77 | d1itua_ | 46 | d1mgta2 | 65 | d2phla1 | 87 |
| 79 | d1kfta_ | 1333 | d1pa2a_ | 3.608 | d1kful2 | 77 | d1kful2 | 46 | d1fo4a6 | 66 | d1b6q__ | 88 |
| 80 | d1gvna_ | 1333 | d1g96a_ | 3.607 | d1bmv22 | 77 | d1df0a2 | 46 | d1ix9a1 | 67 | d1b67a_ | 90 |
| 81 | d1cpq__ | 1333 | d1brfa_ | 3.606 | d1cpq__ | 77 | d1b67a_ | 46 | d1o6wa1 | 68 | d1e0la_ | 91 |
| 82 | d1a04a2 | 1333 | d1pcfa_ | 3.588 | d1gvna_ | 77 | d1jqga2 | 47 | d1h6gb1 | 69 | d1cpq__ | 92 |
| 83 | d1itha_ | 1333 | d1cm8a_ | 3.587 | d1itha_ | 77 | d1gkma_ | 47 | d1e0la_ | 69 | d1m6ba2 | 95 |
| 84 | d1kful2 | 1333 | d1fw4a_ | 3.577 | d1k0ga_ | 102 | d1eera_ | 48 | d1ljya1 | 70 | d1nkd__ | 96 |
| 85 | d1df0a2 | 1333 | d1jjya_ | 3.574 | d1cjaa_ | 102 | d1ihua1 | 49 | d1o0sa2 | 70 | d1mhna_ | 96 |
| 86 | d1dgwa_ | 1333 | d1g8ia_ | 3.558 | d1gjoa_ | 102 | d1gx5a_ | 49 | d1zpda2 | 70 | d1rro__ | 97 |
| 87 | d1g8ia_ | 1333 | d1ihua1 | 3.534 | d1fmk_3 | 102 | d1e9la1 | 50 | d1g8ia_ | 71 | d1b0nb_ | 98 |
| 88 | d1bmv22 | 1333 | d1itua_ | 3.528 | d1muoa_ | 102 | d1b6q__ | 50 | d1vcpa_ | 71 | d1mgta2 | 98 |
| 89 | d1icra_ | 1333 | d1ggza_ | 3.498 | d1atla_ | 102 | d1iwga4 | 50 | d1xbl__ | 71 | d1fo4a6 | 99 |
| 90 | d1fo4a6 | 1333 | d1g0ha_ | 3.496 | d1l0la2 | 102 | d1dgwa_ | 51 | d1ebfa2 | 72 | d1o0sa2 | 101 |
| 91 | d1fvpa_ | 1333 | d1etzb1 | 3.487 | d1ngvb_ | 102 | d1bkza_ | 52 | d1kola2 | 74 | d1itha_ | 102 |
| 92 | d1i5pa3 | 1333 | d1xo1a1 | 3.482 | d1ijba_ | 102 | d1f6fb2 | 52 | d2phla1 | 77 | d1dgsa1 | 103 |
| 93 | d2ebn__ | 1333 | d1db3a_ | 3.474 | d1ls1a2 | 102 | d1nkd__ | 53 | d1g0ha_ | 77 | d1gtoa_ | 104 |
| 94 | d1mira_ | 1333 | d1gtoa_ | 3.472 | d1cp2a_ | 102 | d1b6cb_ | 53 | d4aig__ | 77 | d1p16a1 | 105 |
| 95 | d1n3la_ | 1333 | d1kjqa1 | 3.471 | d1n3la_ | 102 | d1bmv22 | 54 | d1cpq__ | 79 | d1pa2a_ | 106 |
| 96 | d1itua_ | 1333 | d1d9aa_ | 3.458 | d1ms9a2 | 102 | d1gtoa_ | 55 | d1c16a2 | 80 | d1g8ia_ | 107 |
| 97 | d4xiaa_ | 1333 | d1lq9a_ | 3.455 | d1p4ma_ | 102 | d1pls__ | 57 | d1h6ga1 | 80 | d7gata_ | 114 |
| 98 | d3tata_ | 1333 | d1n08a_ | 3.454 | d1etzb1 | 102 | d7gata_ | 59 | d1agx__ | 81 | d1iwga4 | 115 |
| 99 | d1m7ya_ | 1333 | d1id0a_ | 3.444 | d1m98a1 | 102 | d1b06a1 | 60 | d1no1a_ | 82 | d1znf__ | 116 |
| 100 | d1i7da_ | 1333 | d1itha_ | 3.444 | d1iala_ | 102 | d1d5ca_ | 61 | d1m6ba2 | 83 | d4aig__ | 116 |
